# Medical liver biopsy: background, indications, procedure and histopathology

**DOI:** 10.1136/flgastro-2018-101139

**Published:** 2019-03-02

**Authors:** Alexander Boyd, Owen Cain, Abhishek Chauhan, Gwilym James Webb

**Affiliations:** 1 Biomedical Research Centre, University Hospitals Birmingham NHS Foundation Trust and University of Birmingham, Birmingham, UK; 2 Centre for Liver and Gastrointestinal Research, Institute of Immunology and Immunotherapy, University of Birmingham, Birmingham, UK; 3 Liver Unit, University Hospitals Birmingham NHS Foundation Trust, Birmingham, UK; 4 Histopathology, University Hospitals Birmingham NHS Foundation Trust, Birmingham, UK; 5 Hepatology, Cambridge University Hospitals NHS Foundation Trust, Cambridge, UK

**Keywords:** liver, histopathology, liver biopsy

## Abstract

Histological analysis of liver tissue continues to play an important role in modern hepatological practice. This review explores the indications for medical liver biopsy in addition to the procedure itself, potential complications, preparation of tissue and routine staining. A broad selection of histological images is included to illustrate the appearance of liver tissue both in health and in several important diseases.

## Introduction

Liver biopsy continues to play an important role in modern clinical practice. Biopsies can be ‘targeted’ (surgical) at a lesion or ‘non-targeted’ (medical) to allow assessment of the liver parenchyma in general. This review discusses indications for medical liver biopsy, the procedure itself, possible complications, sample preparation, staining and histological findings both in normal liver tissue and in several important diseases. A familiarity with histological appearances can improve understanding of pathophysiology and underpins good clinical decision-making. We have endeavoured to provide high-quality images to support the descriptions given.

## Indications for performing a liver biopsy

The advent of non-invasive tools such as serum biomarkers and elastography has given clinicians additional tools for estimating the severity of fibrosis.^1^ While this has resulted in reduced overall need for liver biopsy, histological analysis of the hepatic parenchyma remains an important investigation.

Broadly, the indications for medical liver biopsy fall into two groups. First, establishing a diagnosis, which includes ruling out other diagnoses or determining the predominant pathology if more than one cause of liver injury is present. Second, liver biopsy allows assessment of disease severity including staging and grading.^2^ Staging pertains to the severity of fibrosis: although non-invasive tests increasingly have a role to play, biopsy is generally considered the ‘gold-standard’ test provided a satisfactory sample is obtained. Grading involves determining the severity of the underlying disease process.

Biopsies are occasionally carried out in acute liver failure. Biopsy in this setting would be determined on a case-by-case basis; recent guidance suggests that it can be considered to rule out infiltrative malignancy, alcohol-related pathology and cirrhosis.^3^

## Performing a liver biopsy

Liver biopsies are usually obtained via a percutaneous approach using ultrasound guidance. Coagulopathy and thrombocytopenia are common problems in patients with liver disease and may pose an unacceptable risk for a percutaneous approach: in these cases, a trans-venous approach can be used instead, avoiding puncture of the liver capsule.^4^

For a trans-venous approach, the internal jugular vein is normally used to introduce a catheter into one of the hepatic veins under fluoroscopic guidance,^5^ and a biopsy needle is then advanced. This approach reduces the risk of significant bleeding, although the core of tissue obtained may be smaller and multiple needle passes needed.^6^ Other reasons for a trans-venous approach include ascites, simultaneous acquisition of a hepatic venogram or pressure studies and obesity or other anatomical considerations where a safe percutaneous puncture site cannot be identified. Biopsies can be obtained laparoscopically, although this is not routine in the UK and is often opportunistic due to the presence of a macroscopically abnormal liver.

In general, use of a 16G or wider cutting needle is recommended for liver biopsy. The presence of 10–12 portal tracts within the specimen is considered sufficient for reliable analysis,^7^ ensuring that architectural relationships between structures are maintained. The length of the biopsy specimen correlates with the number of portal tracts,^8^ and therefore a length of 20–25 mm is preferred. Specimens which are suboptimal in size may result both in diagnostic inaccuracy and in the inaccurate assessment of disease severity.^7^

## Potential complications

Liver biopsies are usually carried out as a day-case admission, with bed rest and basic non-invasive monitoring following the procedure. Overnight admission may be warranted if the patient lives alone. Postprocedural pain is usually mild.^9^ The most significant risk is bleeding—this is often immediate but can present up to 1 week after the procedure.^10^ Other risks include haemobilia, non-hepatic organ puncture, infection and reaction to local anaesthetic.

Severe bleeding following a percutaneous liver biopsy is usually intraperitoneal and haemodynamic instability is common.^7^ A UK-wide audit in 2013 reported a risk of clinically significant bleeding (with drop in blood count, radiological evidence of bleeding and requirement for intervention) of 0.4%. The mortality rate was 0.11%,^11^ with bleeding the cause in all cases. It is worth highlighting that targeted biopsy was linked to a greater risk of major complications than non-targeted biopsy.

## Staining

Liver biopsy specimens are fixed in formalin to form cross-links and prevent degradation. Specimens are then dehydrated and embedded in paraffin before microtome sectioning and mounting on slides. Non–formalin-fixed tissue may be needed for tests such as microbiological analysis or copper quantification studies.^7^ The standard initial stain is H&E,^12^ which stains nuclei blue and cytoplasm and fibrous tissue pink. Other routine stains carried out are listed below in table 1.^13^ Immunohistochemistry is used in particular situations, some of which are mentioned in the relevant sections later in the article.

**Table 1:** Summary of the routine stains used for a medical liver biopsy in addition to H&E

Stain	Material stained	Relevance in liver biopsy
Reticulin	Type III collagen fibres	Useful for assessing gross architecture of the specimen. Condensation of reticulin fibres is seen in areas of recent hepatocellular necrosis and in fibrosis Also useful for highlighting nodular changes in nodular regenerative hyperplasia
Haematoxylin van Gieson*	Type I collagen fibres	Normally only present in portal tracts and around hepatic veins; increased staining seen in fibrosis
Orcein	Hepatitis B surface antigen	May be present in chronic Hepatitis B infection
	Copper-associated protein	Can be increased in any cause of chronic cholestasis and Wilson’s disease
	Elastic fibres	Produced in longstanding fibrosis and can help differentiate this from recent architectural changes caused by necrosis and collapse
Periodic acid–Schiff	Glycogen	Stains hepatocytes which contain abundant glycogen, useful to both detect hepatocytes and show their absence (for example in confluent necrosis)
Periodic acid–Schiff diastase	Mucin Alpha-1 antitrypsin	Following the digestion of glycogen with diastase abnormal polymers of alpha-1 antitrypsin can be seen in cases of alpha-1 antitrypsin deficiency
Perls’ stain	Haemosiderin (ferric iron)	Increased iron deposition is seen in any cause of iron overload

*Other stains such as Sirius red and trichrome may also be used to visualise collagen fibres.

## Histological assessment

In a medical liver biopsy, the normal liver architecture should be maintained unless there are marked morphological changes due to cirrhosis or necrosis. Usually areas of hepatic parenchyma with hepatocytes and sinusoids are seen (‘lobular’ region), which are interspersed with central venules and portal tracts. Portal tracts usually contain a portal vein, a branch of the hepatic artery and a bile duct.^13^

**Figure 1:**
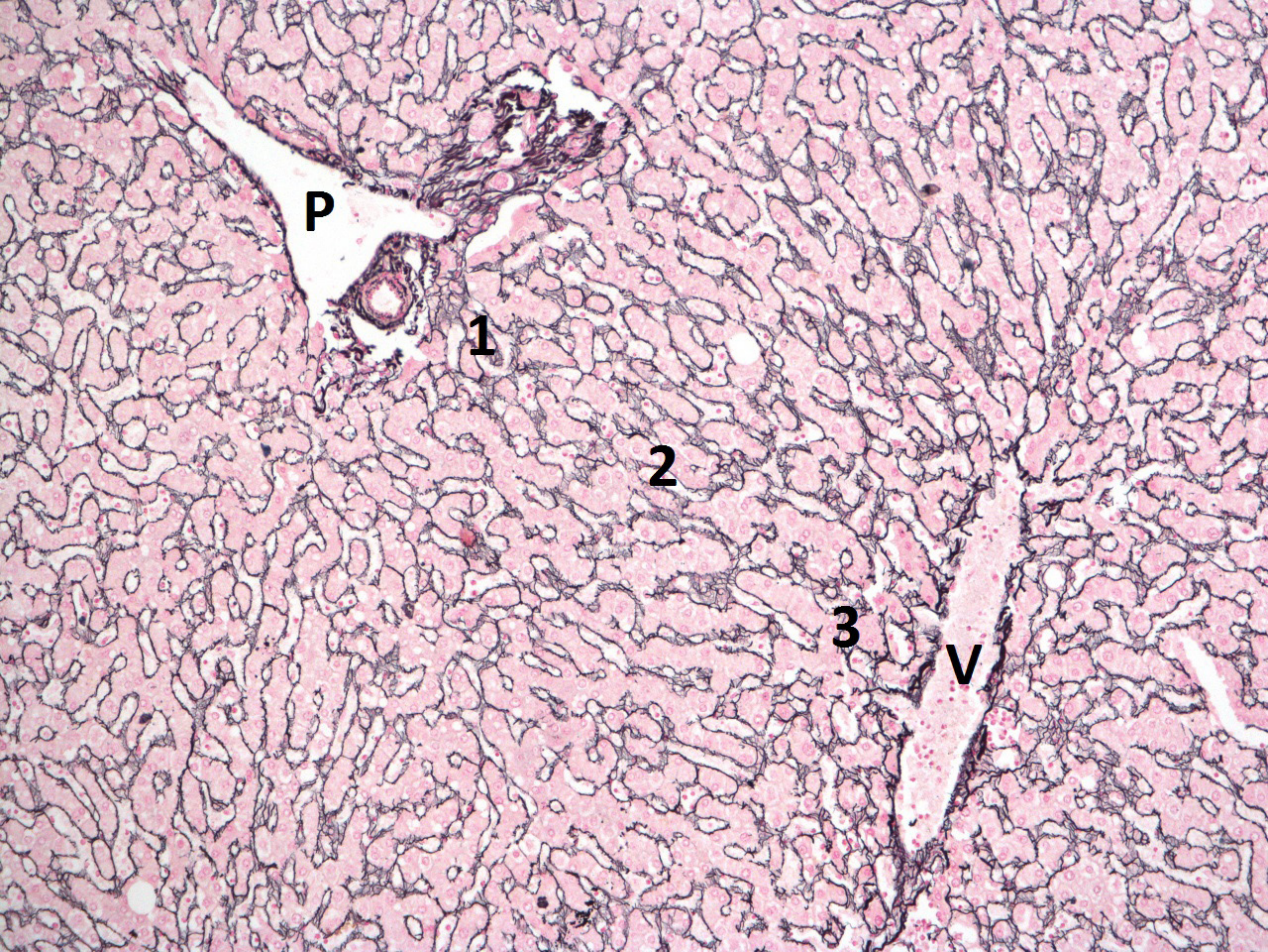
Reticulin stain showing normal liver parenchyma with a portal tract in the top left of the image (P) and a central vein in the bottom right (V). Zones 1–3 are labelled.

Three different ‘zones’ are defined by these structures, although the anatomy is complex: zone 1 is adjacent to the portal tract, zone 3 is closest to the hepatic venule and zone 2 is in between (see figure 1). This arrangement of structures is also called an ‘acinus’.^12^

## Fibrosis

Fibrosis is a dynamic process caused by chronic injury, inflammation and regeneration. This usually begins in a particular area (eg, zone 1 in chronic biliary disease) and extends outwards as thin fibrous septa. These can then extend to adjacent portal tracts or central venules which is known as bridging fibrosis,^12^ a precursor to cirrhosis. As the process advances further, the cirrhotic changes of distortion and nodule formation ensue. The use of stains (eg, HvG and orcein—see table 1) can help to distinguish the presence of long-standing fibrosis, which is associated with the formation of mature elastic fibres, from the collapse of normal architecture which can be seen in acute hepatitis with necrosis.^13^

**Figure 2:**
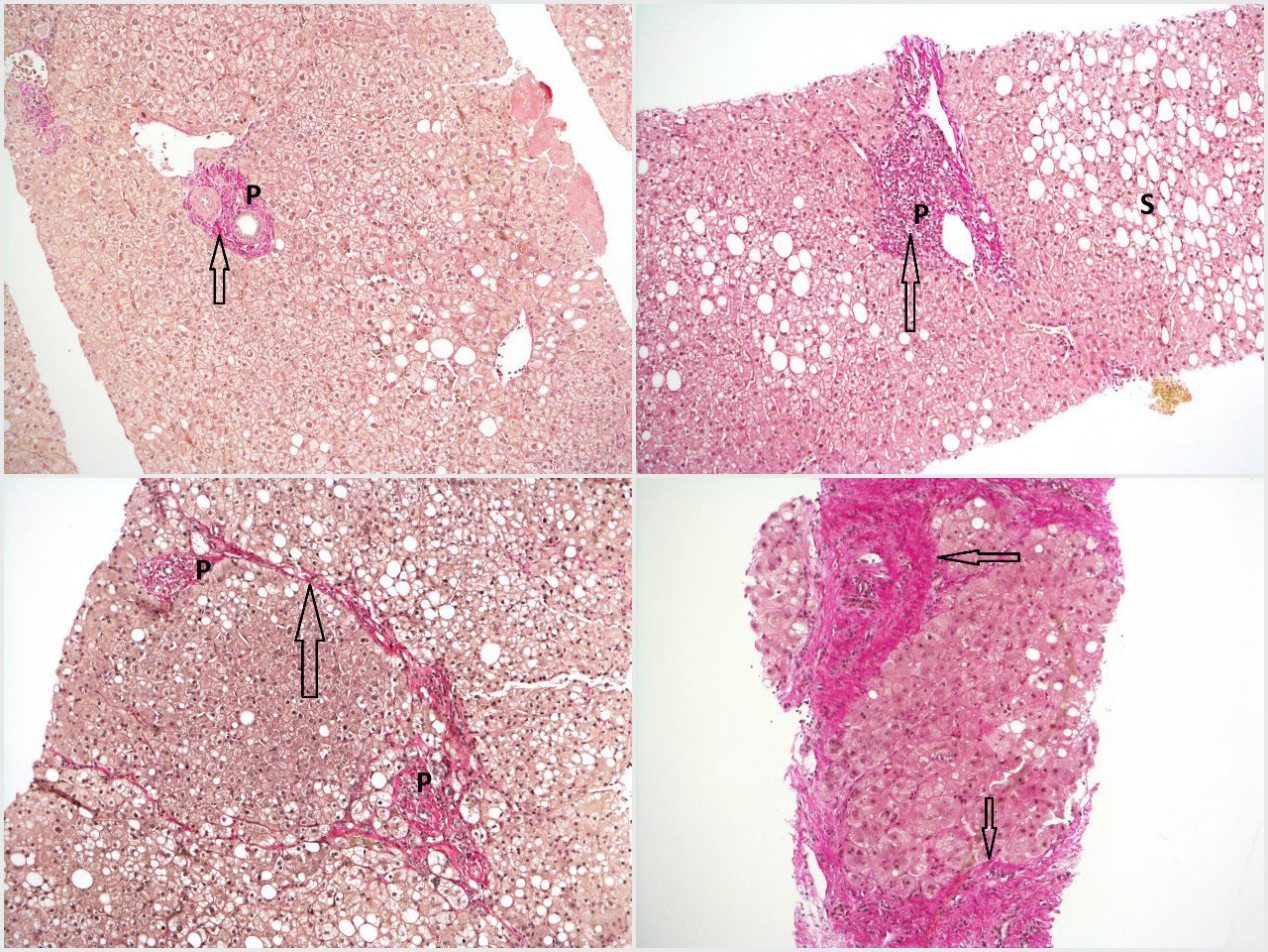
Top left—haematoxylin van Gieson (HvG) stain showing mild zone 3 steatosis without fibrosis, in which collagen fibres (pink–red, arrow) are confined to portal tracts (P). Top right—HvG stain showing steatohepatitis with mild fibrosis in the form of fibrous expansion (arrow) of the portal tract (P). Note the presence of steatosis (S). Bottom left—HvG stain showing steatohepatitis with moderate fibrosis, with thin fibrous bridges (arrow) linking adjacent portal tracts (P). Bottom right—HvG stain showing steatohepatitis with established cirrhosis, with thick bands of fibrosis (arrows) encircling a hepatocyte nodule.

Figure 2 shows the spectrum of fibrosis within the context of fatty liver disease from steatosis without fibrosis to mild fibrosis, moderate fibrosis (with bridging) and established cirrhosis.

## Fatty liver disease

Fatty liver is defined by an abnormal deposition of fat (or steatosis) within more than 5% of hepatocytes^14^—this is best appreciated in figure 2. The two most common causes of fatty liver are excessive alcohol consumption and in association with insulin resistance and the metabolic syndrome (non-alcoholic fatty liver disease, NAFLD). Other causes include drugs, coeliac disease, parenteral nutrition, starvation and HIV. Steatosis alone does not necessarily have any significant sequelae; however, inflammation (steatohepatitis) may result which can lead to fibrosis.

Biopsies are undertaken in both alcohol-related and non–alcohol-related aetiologies of fatty liver disease, although importantly it is usually not possible to tell the two aetiologies apart histologically.^12^ In alcohol-related liver disease, biopsy may be considered in cases of diagnostic uncertainty or to accurately stage the disease.^15^ Liver biopsy used to be recommended in the context of severe alcoholic hepatitis to both confirm the presence of steatohepatitis and rule out other causes of liver injury; however, recent international guidelines and general opinion may be shifting away from this approach.^15 16^

Biopsy in NAFLD is usually undertaken to ascertain whether significant fibrosis is present. Typically, this need arises when biomarkers or elastography suggest advanced fibrosis (these tests perform less well at ‘ruling in’ advanced fibrosis in this context than ‘ruling out’^1^) or are discordant.^17^ The biopsy may also reveal findings of non-alcoholic steatohepatitis (NASH)—a risk factor for disease and fibrosis progression.^18^ It is worth noting that NASH can only be diagnosed with a liver biopsy and describes the presence of steatosis, ballooning and lobular inflammation (with or without fibrosis); less severe histological changes would be termed non-alcoholic fatty liver (NAFL) and confer a lower risk of disease progression.

**Figure 3:**
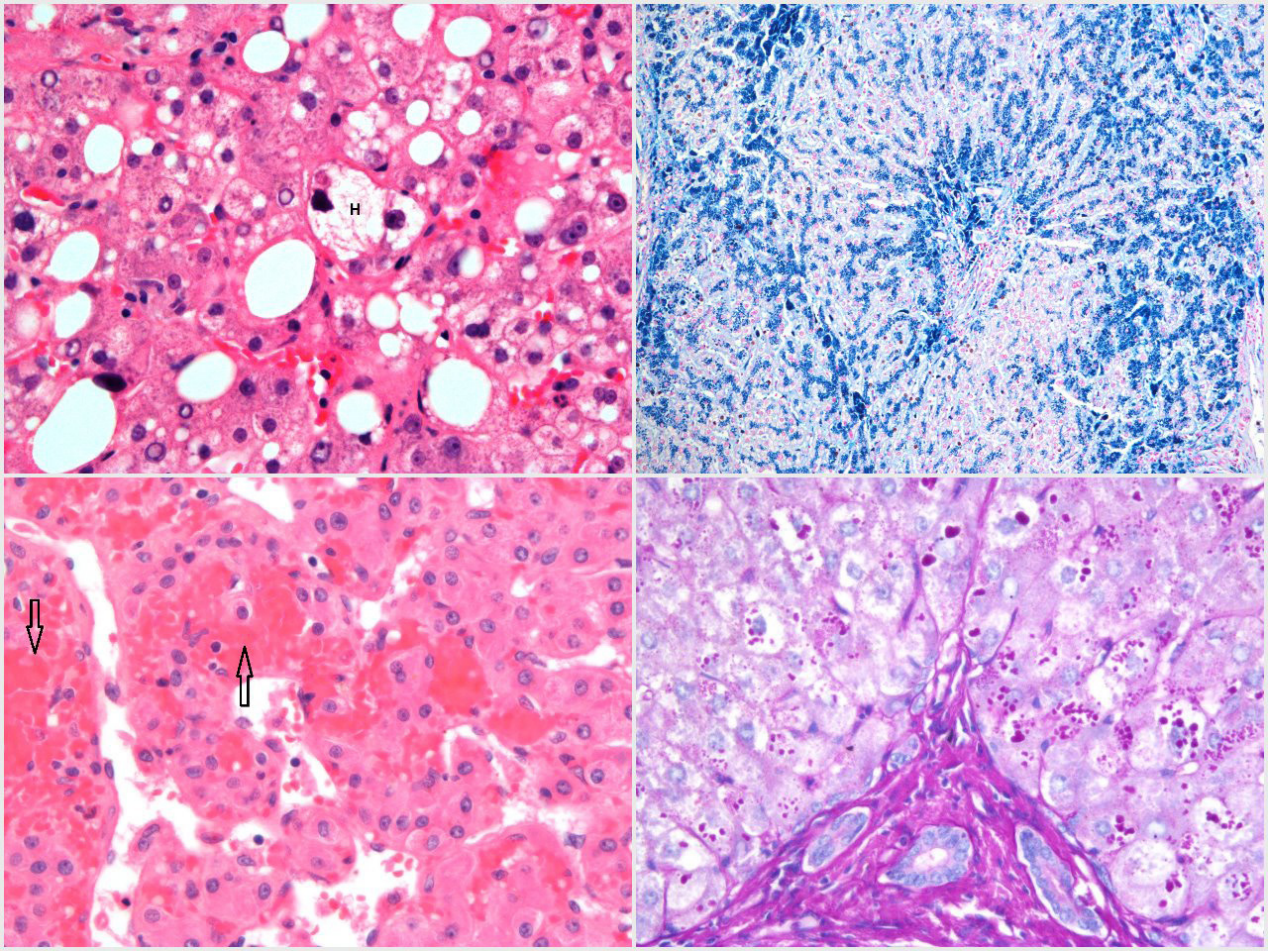
Top left—H&E stain showing scattered large and medium-size droplet steatosis and hepatocyte ballooning in the centre of the field, composed of a hepatocyte (H) with swollen optically clear cytoplasm containing a Mallory-Denk body. Top right—Perls’ stain showing heavy iron deposition in hepatocytes and biliary epithelium (a bile duct is present in the centre of the image) in the context of HFE haemochromatosis. Bottom right—PAS-D stain showing numerous PAS-D-positive globules within hepatocyte cytoplasm, adjacent to a portal tract (bottom centre of image). Globules tend to be concentrated in periportal hepatocytes. Bottom left—H&E stain showing typical changes of Budd-Chiari syndrome with dilated and relatively empty sinusoids, with numerous red blood cells translocated into the space of Disse (arrows).

The inflammation in steatohepatitis is usually more lobular than portal which, if excessive, may signify an additional process (although children/adolescents with NASH can have a more portal distribution).^14^ Mononuclear cells are commonly seen, and neutrophils are perhaps more common in alcoholic steatohepatitis and can circle hepatocytes termed ‘satellitosis’.^12^ Ballooning of hepatocytes is seen, where they swell and increase in size with disruption of the cytoskeleton. Mallory-Denk bodies are typically found within the cytoplasm of ballooned hepatocytes—these are inclusion bodies comprised of cytokeratin with a ‘twisted rope’ appearance.^19^ Immunohistochemical staining for cytokeratin and ubiquitin can be used to highlight Mallory-Denk bodies. Figure 3 and online supplementary figure 1 demonstrate steatohepatitis with ballooning of hepatocytes and Mallory-Denk bodies.

10.1136/flgastro-2018-101139.supp1 Supplementary data

Fibrosis in fatty liver disease usually affects the pericentral (zone 3) region first, extending along sinusoids and around individual hepatocytes in a ‘chicken-wire’ pattern.^20^ Portal fibrosis is generally seen later in the process. Eventually, bridging fibrosis and cirrhosis can ensue.

The NAFLD activity score was designed originally for consistency of reporting in trials; however, it is often used in clinical practice and familiarity with it can be helpful.^21^ The maximum score is 8, with the parameters comprising steatosis (0–3), ballooning (0–2) and lobular inflammation (0–3). The degree of fibrosis is also scored from 1 (mild) to 4 (cirrhosis). A more recent Steatosis/Activity/Fibrosis score uses an algorithmic approach to designate biopsies as ‘No NAFLD’, ‘NAFLD’ or ‘NASH’.^22^

## Hepatitis

### Acute hepatitis

**Figure 4:**
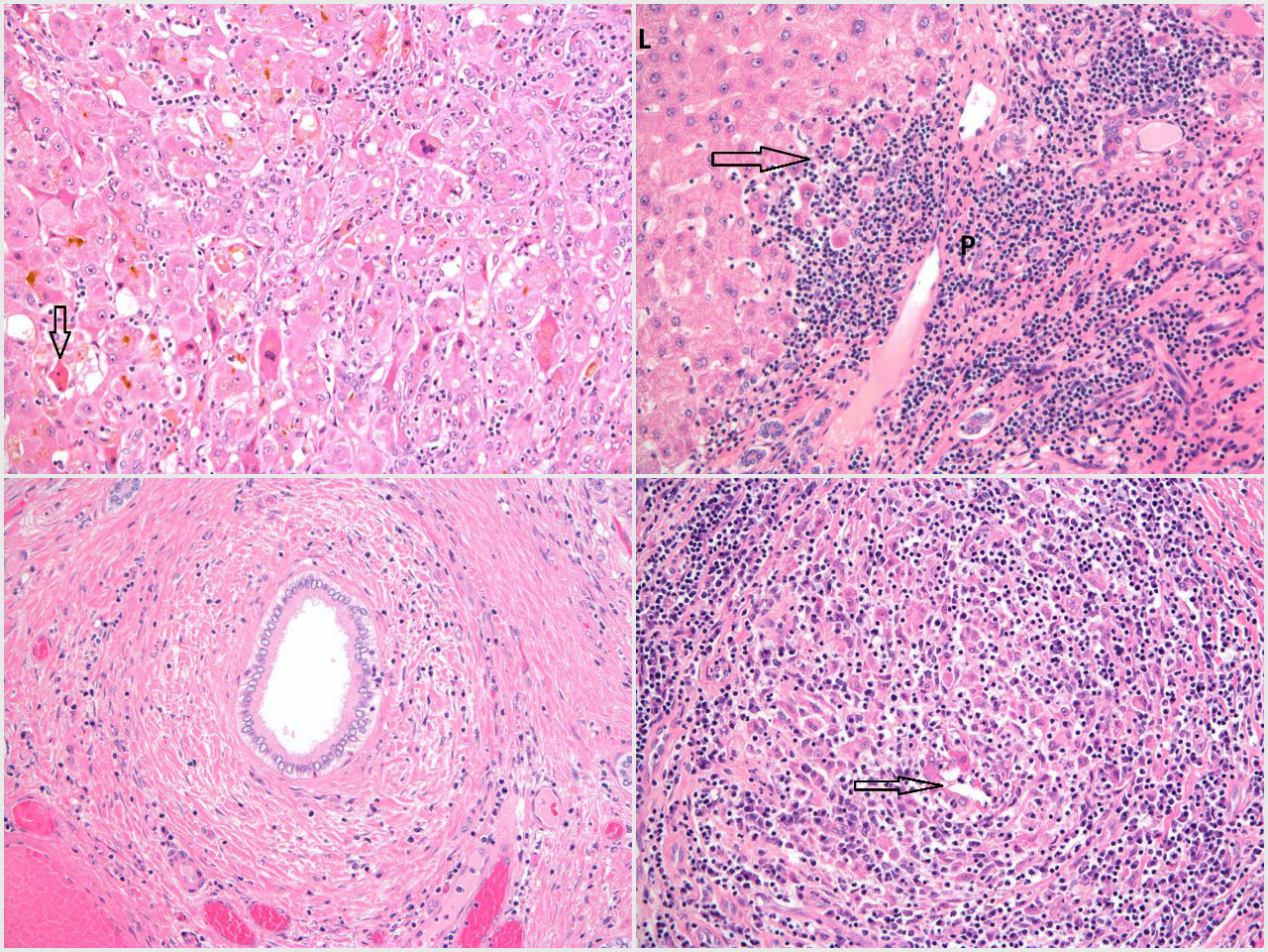
Top left—H&E stain showing acute hepatitis with lobular disarray and associated lymphocytic inflammation, acidophil body formation (arrow) and bilirubinostasis. Top right—H&E stain showing features of chronic hepatitis in the context of chronic hepatitis B infection. There is a moderately dense portal infiltrate comprising predominantly lymphocytes, showing conspicuous interface activity (arrow). L, lobule; P, portal tract. Bottom right—H&E stain showing the classical changes of primary biliary cholangitis with a florid granulomatous inflammatory bile duct lesion. The residual damaged duct is seen in the centre of the image (arrow). Bottom left—H&E stain showing concentric fibrosis surrounding an inflamed bile duct, a typical change of primary sclerosing cholangitis.

Acute hepatitis usually causes a lobular pattern of inflammation. The inflammation can be mild with minor infiltrates and spotty necrosis of single hepatocytes, or in severe cases cause widespread necrosis with architectural disturbance (lobular disarray) or collapse^12^ (see figure 4).

Histologically determining the cause can be difficult: possibilities include acute viral infections, drug-induced or toxin-induced hepatitis, an acute presentation of autoimmune hepatitis (AIH) or ‘seronegative’ hepatitis. Serology is generally helpful in the diagnosis of viral infections. The presence of an eosinophilic inflammatory infiltrate may support drug-induced injury, although eosinophils are not specific and can be seen in other conditions including AIH.^23^ Factors supporting the diagnosis of AIH include a plasma cell–rich infiltrate^12^ and positive immunology.

### Chronic hepatitis

Chronic hepatitis usually results in a portal distribution of inflammation, with spill-over into the adjacent liver parenchyma (interface hepatitis). A similar differential exists as for acute hepatitis, namely viral infections, drug-induced or toxin-induced injury, and AIH. A number of other diseases may be associated with portal and periportal inflammatory changes, which can resemble those seen in chronic viral hepatitis or AIH. Examples include chronic biliary diseases such as primary biliary cholangitis (PBC) and primary sclerosing cholangitis (PSC), fatty liver disease and some metabolic diseases (eg, Wilson’s disease and alpha-1 antitrypsin deficiency). In most of these cases, the presence of additional histological features points to an alternative diagnosis.

#### Viral hepatitis

Typically, chronic hepatitis B infection causes histological activity in different phases in the natural history of the disease, hence a recent change in terminology to either chronic hepatitis or chronic infection in association with e antigen status.^24^ Orcein staining can demonstrate surface antigen expression, and the degree of activity can influence treatment decisions. Figure 4 and online supplementary figure 2 demonstrate chronic hepatitis due to hepatitis B infection. Hepatitis C can be associated with steatosis and bile duct injury in addition to chronic hepatitis.^12^ Elastography now has a well-established role in the assessment of fibrosis in this context.^1^

10.1136/flgastro-2018-101139.supp2 Supplementary data

#### Autoimmune hepatitis

AIH is characterised by interface hepatitis, but varying degrees of lobular hepatitis can be present. The presence of plasma cells in the infiltrate is suggestive of this diagnosis. The clustering of regenerating hepatocytes in areas of fibrous portal expansion is called a ‘rosette’^23^ (see online supplementary figure 3). Biliary changes are not routinely seen in AIH. Liver biopsy plays an important role in AIH: establishing the diagnosis, grading disease activity and staging the level of fibrosis.^25^

10.1136/flgastro-2018-101139.supp3 Supplementary data

## Biliary disease

### Primary biliary cholangitis

PBC can be diagnosed confidently with biochemical cholestasis and supportive immunology (anti-mitochondrial antibody positivity or specific ANAs), and hence liver biopsy is not a prerequisite for diagnosis. Liver biopsy may be required to establish a diagnosis of AMA-negative PBC in cases of unexplained intrahepatic cholestasis and can be useful if there are concerns about an AIH/PBC overlap syndrome. It is worth noting that accurately defining overlap syndromes can be difficult.^26^

The key finding is a portal inflammatory infiltrate with a granulomatous component centred on small bile ducts (see figure 4). This is associated with injury to the biliary epithelial cells and disruption of the bile duct basement membrane.^12^ Small bile ducts are progressively lost (ductopenia).^27^ Periportal fibrosis then ensues and can progress to cirrhosis.

### Primary sclerosing cholangitis

The histological changes of PSC are variable, and extrahepatic bile ducts are not present in needle liver biopsy specimens. However, small-duct PSC can be diagnosed histologically and some changes may be identified reflecting biliary obstruction from downstream large bile duct strictures. Portal inflammation can be present but is usually less pronounced than in PBC.^12^ Fibrous obliteration of bile ducts can occur; the classical lesion is a concentric ‘onion skin’ fibrosis around an affected duct^28^ (see figure 4). In contrast to PBC, granulomas are not typically seen.

## Metabolic liver disease

### Hepatic siderosis

Hepatic iron deposition can result from iron overload states due to any cause. The most common inherited cause is HFE haemochromatosis. Other conditions causing iron overload include alcohol-related liver disease, non-HFE haemochromatosis, ferroportin disease and repeated transfusion.^29^ Liver biopsy is sometimes undertaken in HFE haemochromatosis to determine the stage of fibrosis, although elastography can often achieve this. In cases of suspected iron overload, liver biopsy (and MRI) can be used to confirm or refute significant hepatic siderosis which can aid diagnosis.^30^

Accumulation of iron is directly toxic to hepatocytes and acts as a stimulus for collagen production.^12^ Portal tracts may show varying degrees of inflammation and iron can be deposited in biliary epithelium. Perls’ (Prussian blue) stains ferric iron deposits (see figure 3).

### Wilson’s disease

Liver biopsy can be undertaken to aid diagnosis of Wilson’s disease and quantitative copper assays performed.^31^ Wilson’s disease can present acutely with liver failure: biopsies undertaken in this scenario typically show hepatocyte degeneration and parenchymal collapse—interestingly, cirrhosis is often present.^12^ In less acute presentations, histological changes can be very varied. As changes progress, appearances may mimic chronic hepatitis with portal inflammation, interface hepatitis and fibrosis.^32^ Importantly, increased copper deposits can be seen in cholestasis from any cause.^12^

### Alpha-1 antitrypsin deficiency

Alpha-1 antitrypsin deficiency can present with chronic liver disease in a small proportion of patients. It is often concomitant with another cause of liver disease. Liver biopsy may reveal mild portal inflammation or simply non-specific fibrotic changes.^12^ The periodic acid–Schiff (PAS) stain, in combination with diastase to digest glycogen (PAS-D), will stain abnormal globules of defective alpha-1 antitrypsin within hepatocytes (see figure 3).

## Vascular liver disorders

### Hepatic venous outflow obstruction

Liver biopsy is not generally required in the work-up of Budd-Chiari syndrome, as the confirmation of venous outflow obstruction is usually made via hepatic venography. However, biopsy can be carried out during venography to clarify the degree of fibrosis. Occlusion of the hepatic venules can be seen, in addition to sinusoidal dilatation and adjacent hepatocyte atrophy.^33^ Erythrocytes may be translocated to the space of Disse (see figure 3). Congestive hepatopathy due to heart failure or constrictive pericarditis can give similar appearances.

### Obliterative portal venopathy (idiopathic non-cirrhotic portal hypertension)

The nomenclature surrounding non-cirrhotic portal hypertension (NCPHT) is confusing. There is a range of conditions that can cause portal hypertension in the absence of significant fibrosis. However, NCPHT is usually taken to mean idiopathic NCPHT. This is a disease associated with obliterative/sclerotic changes to small portal vein branches, and occurs by definition in the absence of extrahepatic portal vein thrombosis and significant hepatic fibrosis.^34^ Nodular regenerative hyperplasia is one particular histological appearance associated with the condition (see online supplementary figure 4).

10.1136/flgastro-2018-101139.supp4 Supplementary data

## Transplantation

Liver biopsy plays an important role in the management of liver allograft recipients, where it is often performed due to graft dysfunction.

Graft dysfunction in the early postoperative period has a variety of causes; biopsy may be helpful to determine the specific diagnosis or indicate the dominant insult. Changes of preservation-reperfusion injury are commonly seen and include microvesicular steatosis, foci of lobular neutrophilic inflammation, cholestasis, hepatocyte ballooning and necrosis.^35^ Acute cellular rejection (ACR) usually occurs in the first month post-transplant. Histologically, a portal pattern of injury occurs with a mononuclear inflammatory infiltrate and inflammation of bile ducts.^35^ Subendothelial inflammation of portal and hepatic venules is also seen (see online supplementary figure 5). Zone 3 necroinflammatory changes reflect greater severity, with the Banff criteria used for grading.^36^ Acute antibody-mediated rejection (AMR) may be associated with histological changes resembling those found in biliary obstruction^37^; positive C4d staining may support a diagnosis of AMR^38^ and serum can be analysed for donor-specific antibodies. Other causes of early graft dysfunction include drug-induced liver injury and acute hepatitis due to viral infections such as cytomegalovirus. Vascular and biliary problems are also common causes of graft dysfunction, usually diagnosed by imaging.

10.1136/flgastro-2018-101139.supp5 Supplementary data

Graft dysfunction in the late postoperative period may be due to viral infections, drug-induced injury, recurrent disease, ‘de-novo’ disease and rejection. Imaging remains important to assess the graft vasculature and biliary tree. Chronic rejection usually results from non-resolving or repeated episodes of ACR; indeed, changes of both ACR and chronic rejection can coexist.^39^ Histological changes of chronic rejection are potentially irreversible and can lead to graft loss in severe cases.^40^ There may be improvement with augmented immunosuppression. The key finding in chronic rejection is ductopenia (without the active portal inflammation seen in ACR) and loss of small branches of the hepatic artery, in addition to varying degrees of perivenular fibrosis^39^ (see online supplementary figure 6).

10.1136/flgastro-2018-101139.supp6 Supplementary data

## Future direction

While patterns of disease and the indications for liver biopsy may be changing, histological analysis of liver tissue seems likely to remain an important investigation. Perhaps one future direction where liver biopsy may evolve is automated fibrosis assessment, with computer analysis demonstrated to improve scoring accuracy and outperform collagen proportionate area assessment in recent studies.^41 42^ As a final point, it is worth remembering that it is imperative to include relevant clinical details for the histopathologist so they can give an informed opinion, and clinicopathological correlation is always of paramount importance.

## Glossary

The following words appear commonly in histology reports (table 2).

**Table 2:** Glossary of common terms and phrases appearing in liver histology reports

Apoptotic body (also known as Councilman or acidophil body)	Hepatocyte undergoing a ‘programmed’ cell death in response to an insult, stains pink on H&E
Ballooning	A process associated with degenerating hepatocytes where they swell in size up to twofold or threefold with a ‘wispy’ clear cytoplasm often containing Mallory-Denk bodies; associated with steatohepatitis and cholate stasis
Bridging fibrosis	Fibrosis extending between adjacent portal tracts or between a portal tract and an adjacent hepatic venule; precursor to cirrhosis
Bilirubinostasis	Impaired flow of bile through the biliary system; abnormal amounts of bile can be seen within hepatocytes or bile canaliculi
Ductopenia	Reduced numbers of visible bile ducts; can be seen as part of PBC and PSC or can be idiopathic or drug-induced (vanishing bile duct syndrome)
Interface hepatitis (previously called piecemeal necrosis)	Inflammation seen in the border between the portal tracts and the liver parenchyma; a feature of chronic hepatitis
Kupffer cell	Star-shaped macrophages found lining sinusoids
Lobule/lobular region	A region containing hepatocytes and sinusoids found between portal tracts and hepatic venules
Mallory-Denk body	Inclusions found within the cytoplasm of ballooned hepatocytes. Composed of cytokeratin filaments, they have a ‘twisted-rope’ appearance
Onion-skin fibrosis	Concentric fibrosis around a bile duct; seen in PSC
Portal tract	Structure containing a branch of the hepatic artery and portal vein and a bile duct
**​**Satellitosis	Inflammatory cells (neutrophils) surrounding a hepatocyte; seen in alcoholic hepatitis
Sinusoid	A sinusoidal blood vessel separated from hepatocytes by the space of Disse; location of mixing of portal and arterial blood
Steatosis	Abnormal fat deposition within hepatocytes (>5%)

PBC, primary biliary cholangitis; PSC, primary sclerosing cholangitis.

10.1136/flgastro-2018-101139.supp7 Supplementary data
