# Supplementary figures and images for: Medical liver biopsy: background, indications, procedure and histopathology
Source: Frontline Gastroenterol. 2019 Mar 2;11(1):40–7. doi: 10.1136/flgastro-2018-101139 (PMC6914302; doi:10.1136/flgastro-2018-101139)

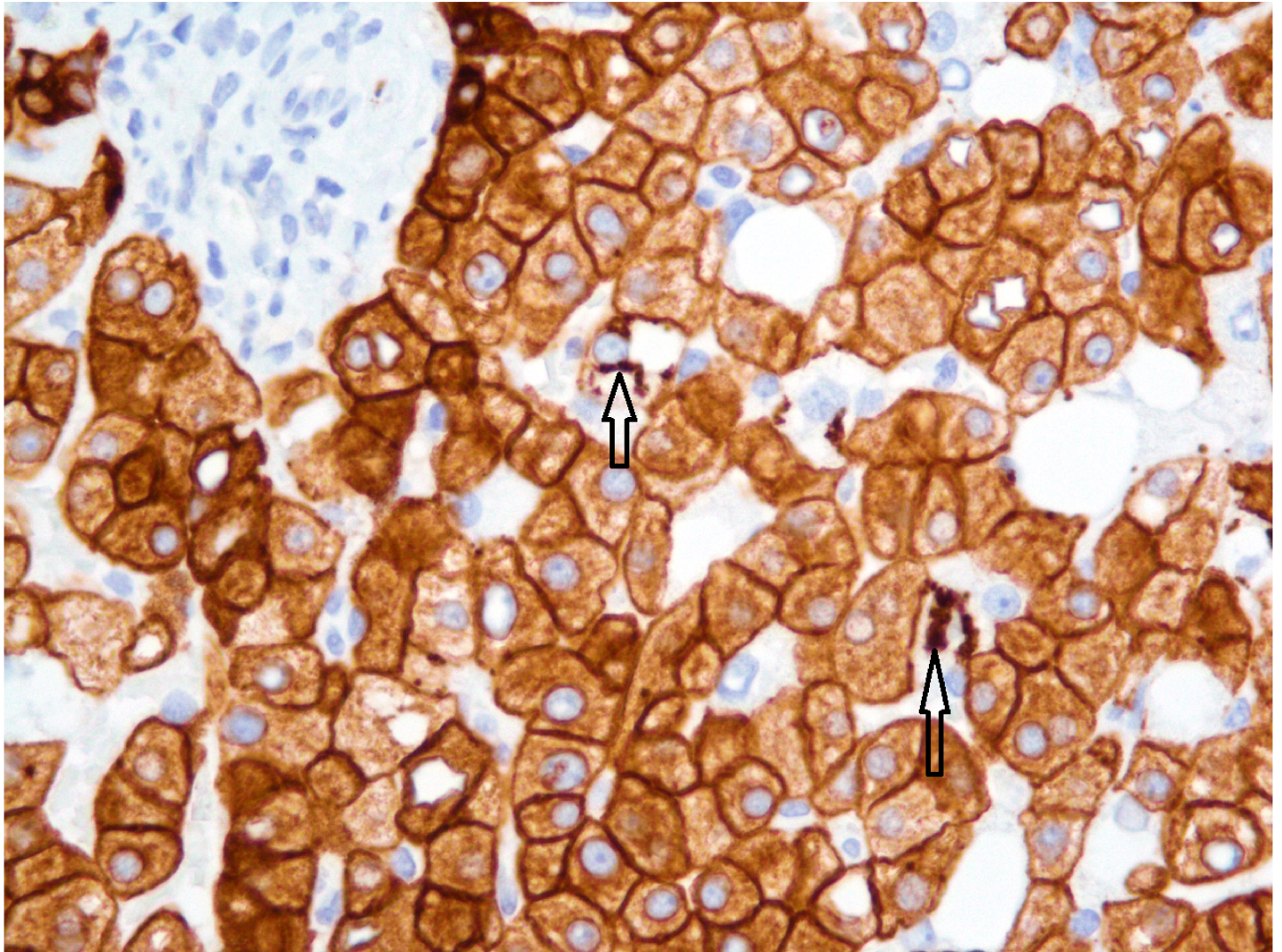

Supplement: Supplementary data [file flgastro-2018-101139supp001.pdf]

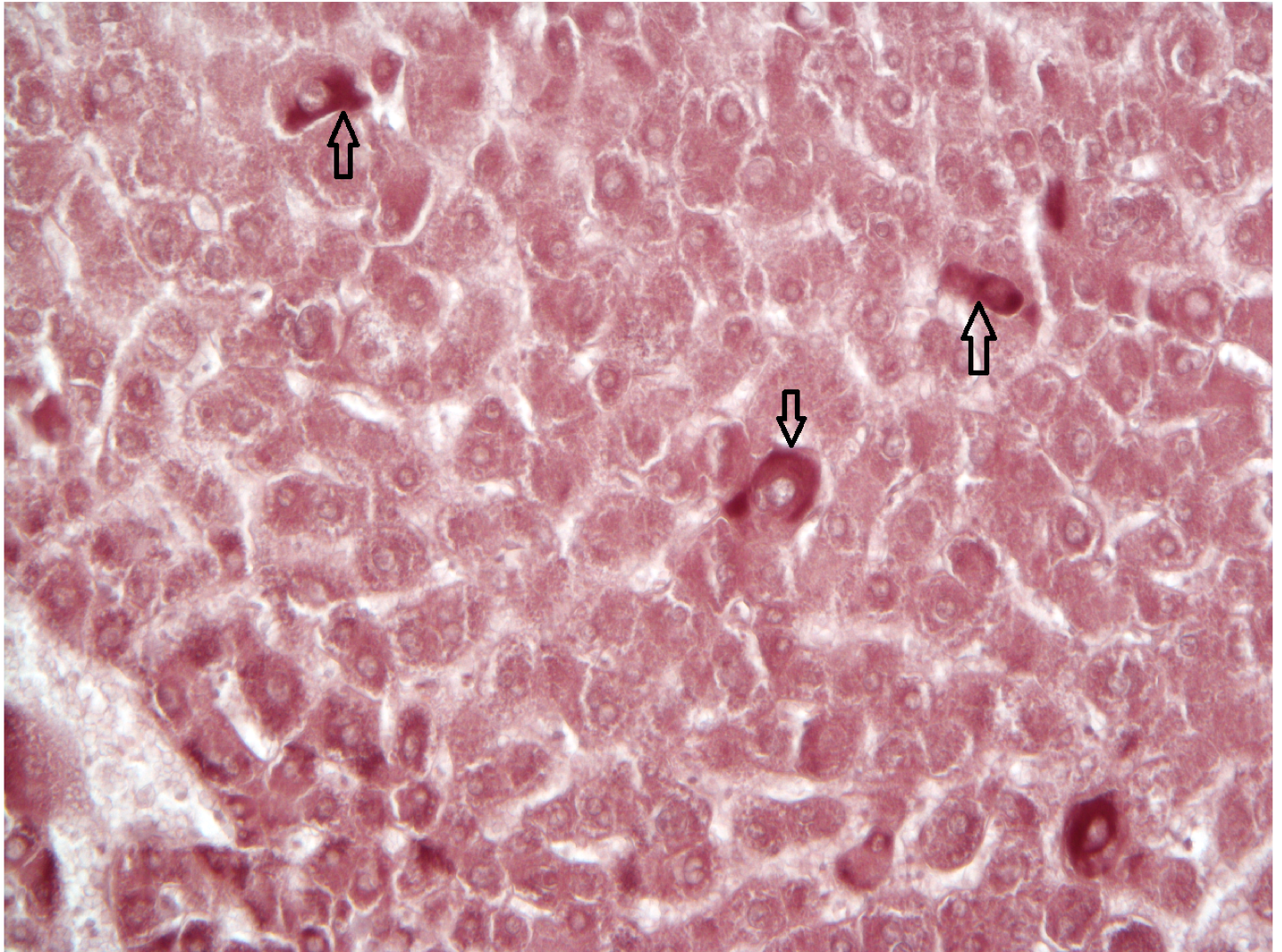

Supplement: Supplementary data [file flgastro-2018-101139supp002.pdf]

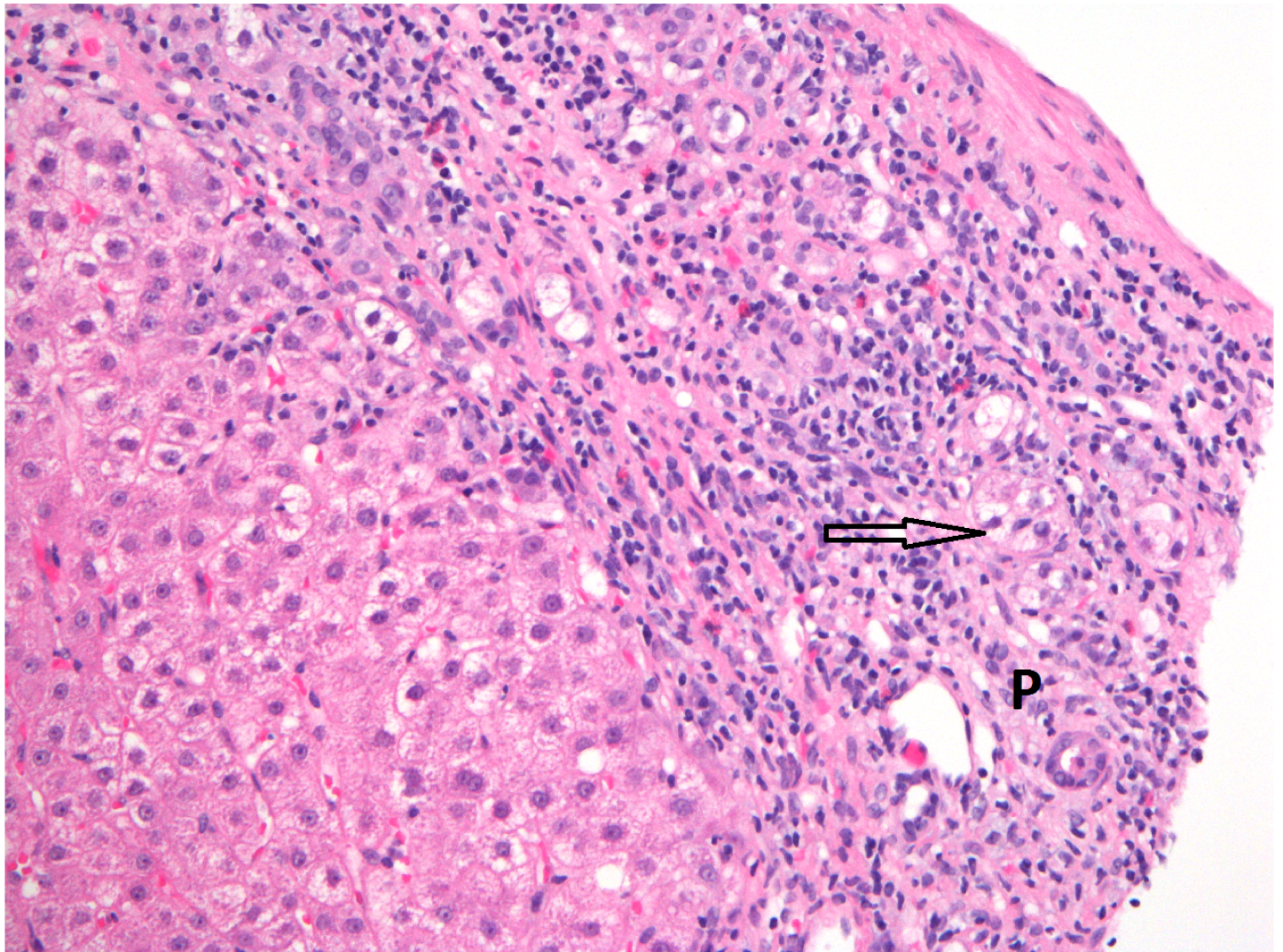

Supplement: Supplementary data [file flgastro-2018-101139supp003.pdf]

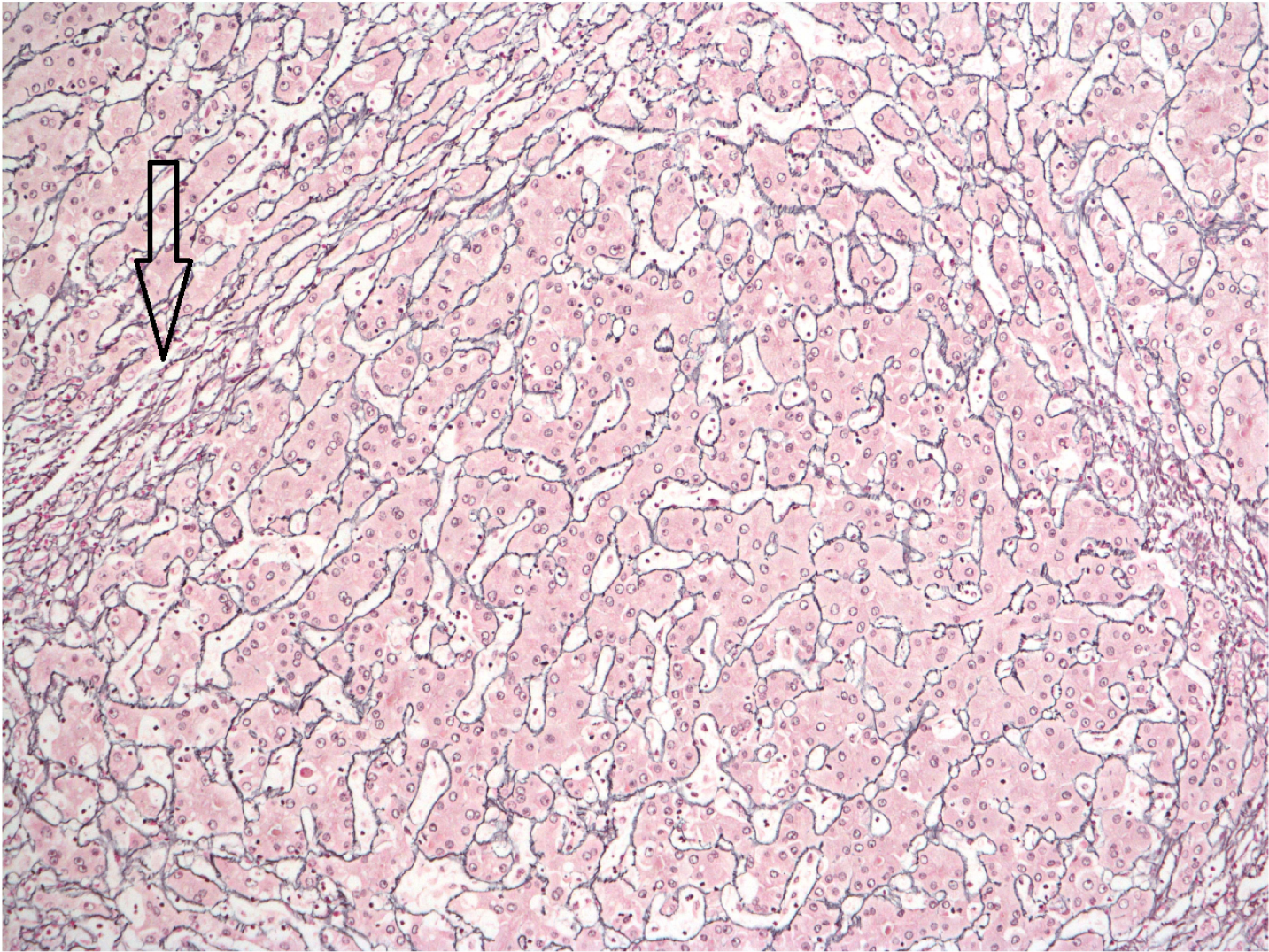

Supplement: Supplementary data [file flgastro-2018-101139supp004.pdf]

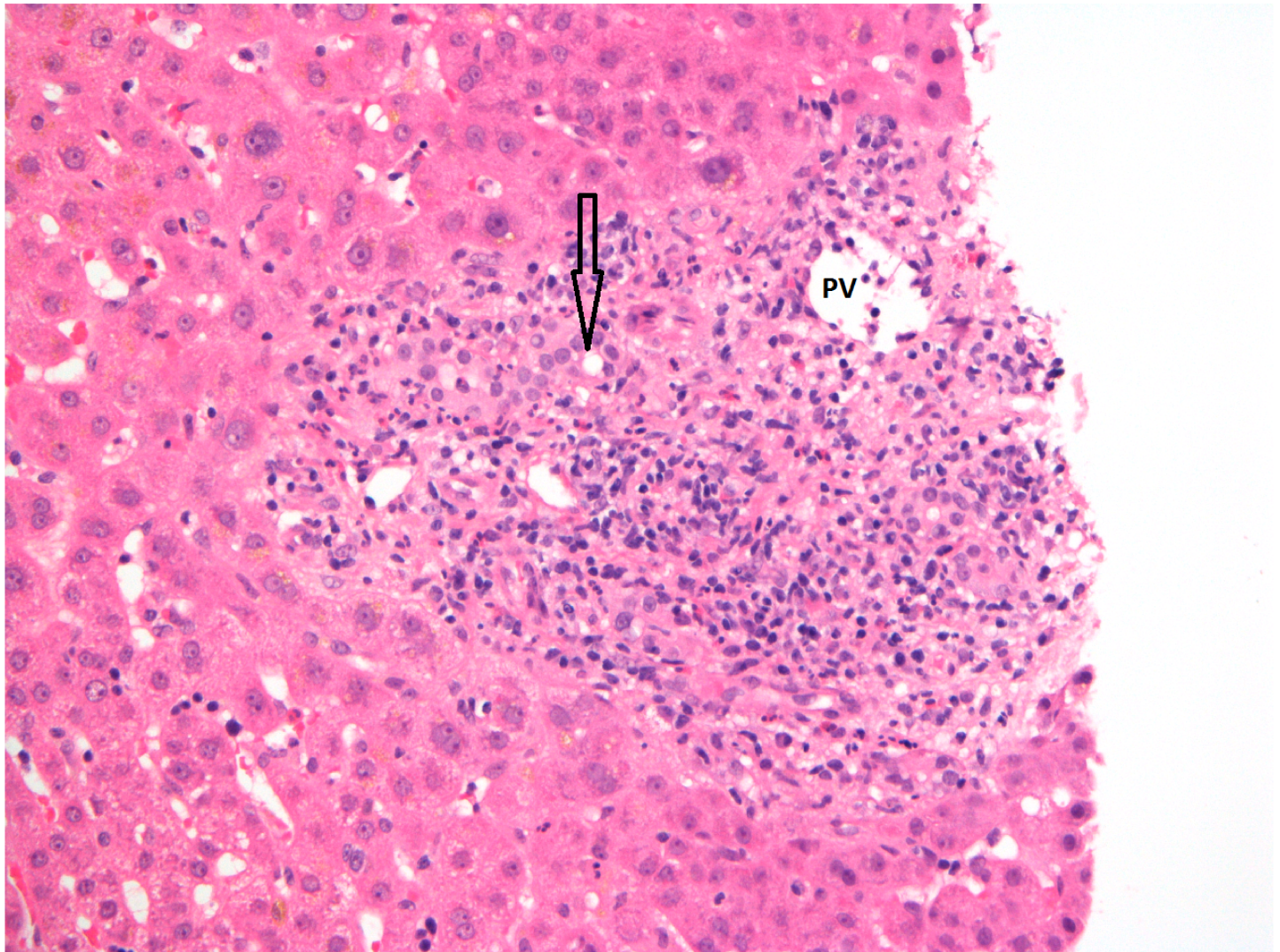

Supplement: Supplementary data [file flgastro-2018-101139supp005.pdf]

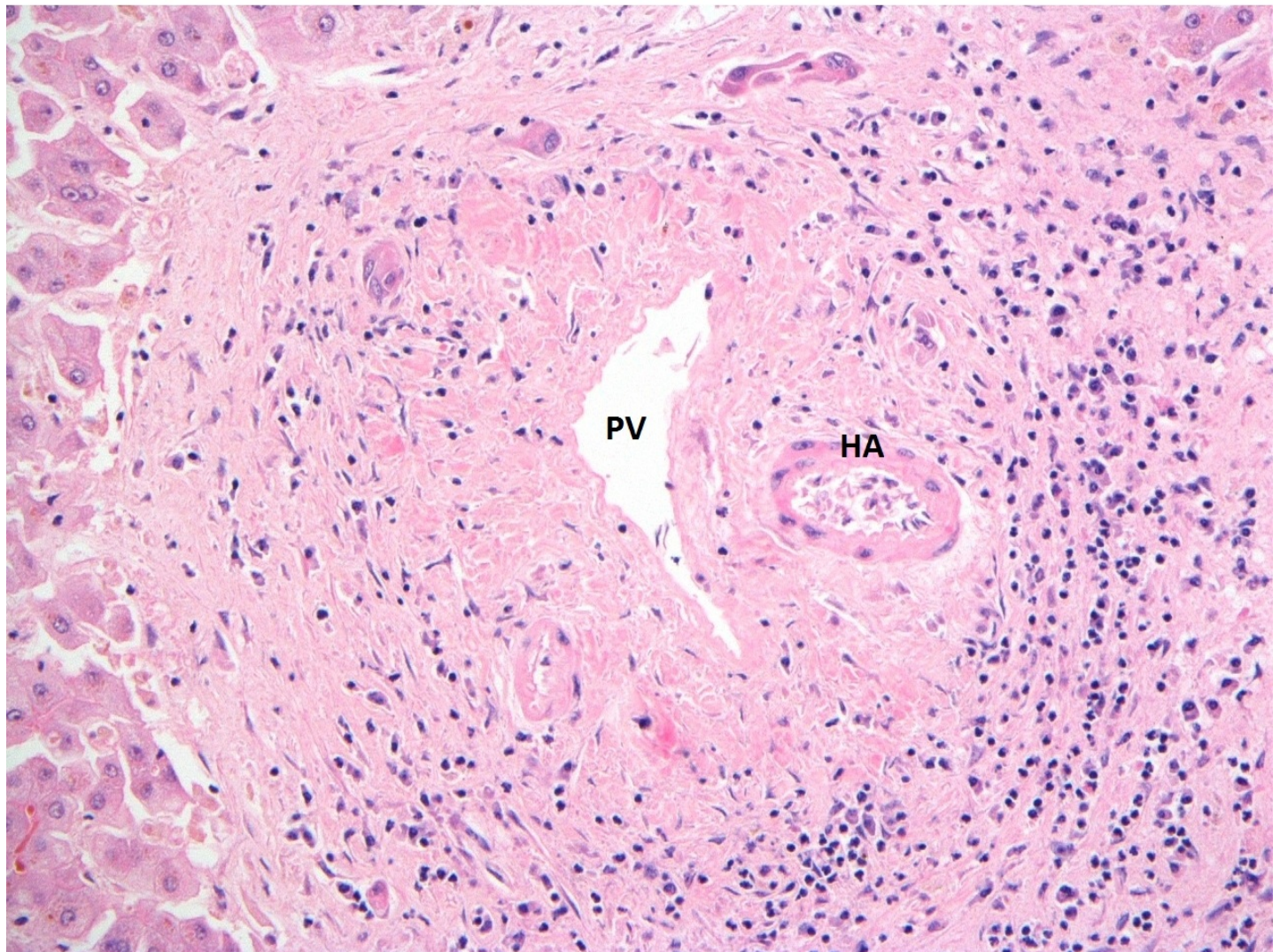

Supplement: Supplementary data [file flgastro-2018-101139supp006.pdf]
