# Supplementary material for: Medical liver biopsy: background, indications, procedure and histopathology
Source: Frontline Gastroenterol. 2019 Mar 2;11(1):40–7. doi: 10.1136/flgastro-2018-101139 (PMC6914302; doi:10.1136/flgastro-2018-101139)
Supplement: Supplementary data [file flgastro-2018-101139supp007.pdf]

Supplementary Figure legends:

Supplementary Figure 1: cytokeratin 8/18 immunohistochemistry stain confirming the presence of Mallory-Denk bodies (arrows) within ballooned hepatocytes. Normally these keratins are diffusely present in the hepatocyte cell membrane and cytoplasm, but during ballooning this staining pattern is lost, and instead the staining of dense cytoplasmic protein aggregates is seen.

Supplementary Figure 2: orcein stain showing the presence of Hepatitis B surface antigen (arrows).

Supplementary Figure 3: H&E stain showing a dense portal infiltrate comprising lymphocytes, plasma cells and eosinophils in the context of AIH. There is interface activity and evidence of hepatocyte "rosetting" (arrow). P = portal tract.

Supplementary Figure 4: reticulin stain showing the appearances of NRH. The parenchyma has a nodular appearance with a band of atrophic hepatocyte plates (arrow) seen at the periphery.

Supplementary Figure 5: H&E stain showing the features of moderate ACR in an early post-transplant biopsy. There is a mixed, moderately dense portal infiltrate comprising lymphocytes, eosinophils, neutrophils and plasma cells. The bile duct (arrow) is inflamed and there is endothelitis of the portal vein branch (PV) with "lifting off" of the endothelial cells.

Supplementary Figure 6: H&E stain showing features of chronic rejection in a late post-transplant biopsy. The portal tract is missing a bile duct (PV = portal vein, HA = hepatic artery). In addition to ductopenia there is mild portal inflammation without endothelitis.
